# 1.G. Workshop: The German National Cohort (NAKO): Design, current state, and first results

**DOI:** 10.1093/eurpub/ckac129.025

**Published:** 2022-10-25

**Authors:** Organised by: NAKO e.V. (Germany), Chair persons: Tobias Pischon (Germany), Lilian Krist (Germany)

## Abstract

This workshop aims to give an overview of design, methods and first results of the largest cohort study in Germany: the German National Cohort (NAKO).This organized session will consist of one overview presentation and three presentations on selected topics: mental disorders, COVID-19, and Magnetic Resonance Imaging (MRI) in the NAKO. NAKO is a multidisciplinary, population-based cohort study that provides a central resource for population-based epidemiologic research. NAKO aims to investigate the development and aetiology of diseases, identify risk factors and enhance early detection and prevention of diseases with a focus on diabetes, cancer, cardiovascular, pulmonary, neurological, psychiatric, and infectious diseases. Between 2014 and 2019, overall 205,415 persons aged 20-74 years were recruited and examined at 18 study centres across Germany. During their visit to the study centre, they participated in a face-to-face interview, completed self-administered, computer-based questionnaires, underwent a battery of biomedical examinations, and provided various biosamples. In addition, whole-body Magnet Resonance Imaging (MRI) was performed on 30,861 participants using dedicated 3 Tesla MRI scanners at 5 study centres. The whole-body MRI protocol focuses on brain and cardiac structures, musculoskeletal system and body fat distribution. In 4-5 year intervals, all study participants are re-invited for examinations at the study centres. The programme for the first re-examination (including MRI scanning) was similar to the baseline programme. Thereby, longitudinal information on changes in risk factor profiles and in vascular, cardiac, metabolic, neurocognitive, pulmonary and sensory function is collected. Since October 2018, 77,896 participants have been re-examined, including 11,382 with additional MRI examination. A supplemental COVID-19 questionnaire collected data on 161,849 participants of NAKO during the first COVID-19-related lockdown in Spring 2020. This survey started on 30 April 2020 and ended on 30 June 2020. The questionnaire included questions on general state of health, Sars-CoV-2 symptoms and test results, and on changes in behavioural, psychosocial and socioeconomic factors as well as social contacts and occupational situation during the pandemic. Thus, psychological and socioeconomic effects of the pandemic situation can be addressed. Moreover, questions on pandemic-related aspects including the history of infection, severity and long-term health impacts of COVID-19 were added to the regular study programme in the study centres as of July 2021. The longitudinal design of NAKO provides the unique opportunity to compare the participants’ situation before and during the pandemic. The presentations describe the main design of the NAKO and exemplary results of main research questions, e. g., on mental health, association of occupational factors with COVID-19, and MRI findings.

**Key messages:**

• The workshop introduces the design and collected data of the NAKO to foster collaboration between scientists, enabling further harmonization of data collection between large cohort studies.

• The workshop aims to facilitate (future) joint scientific exploitation of this unique epidemiological resource of population-based data.

